# Supplementary material for: aPKC and F-actin dynamics promote Hippo pathway polarity in asymmetrically dividing neuroblasts
Source: Biol Open. 2026 Mar 9;15(3):bio062356. doi: 10.1242/bio.062356 (PMC13035065; doi:10.1242/bio.062356)
Supplement: Supplementary information [file biolopen-15-062356-s1.pdf]

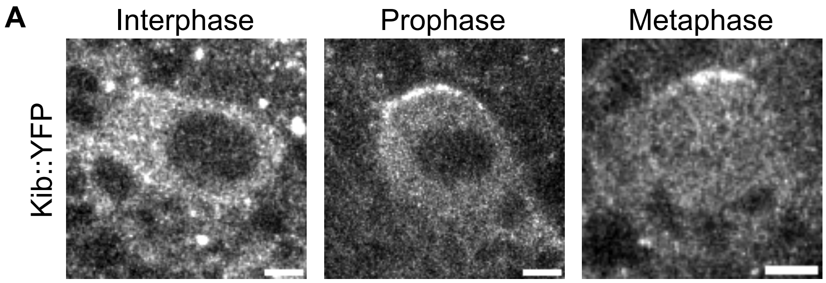

Fig. S1. Endogenously-expressed Kibra polarizes in living neuroblasts. A. Interphase, prophase, and metaphase images of neuroblasts endogenously expressing Kib::YFP.

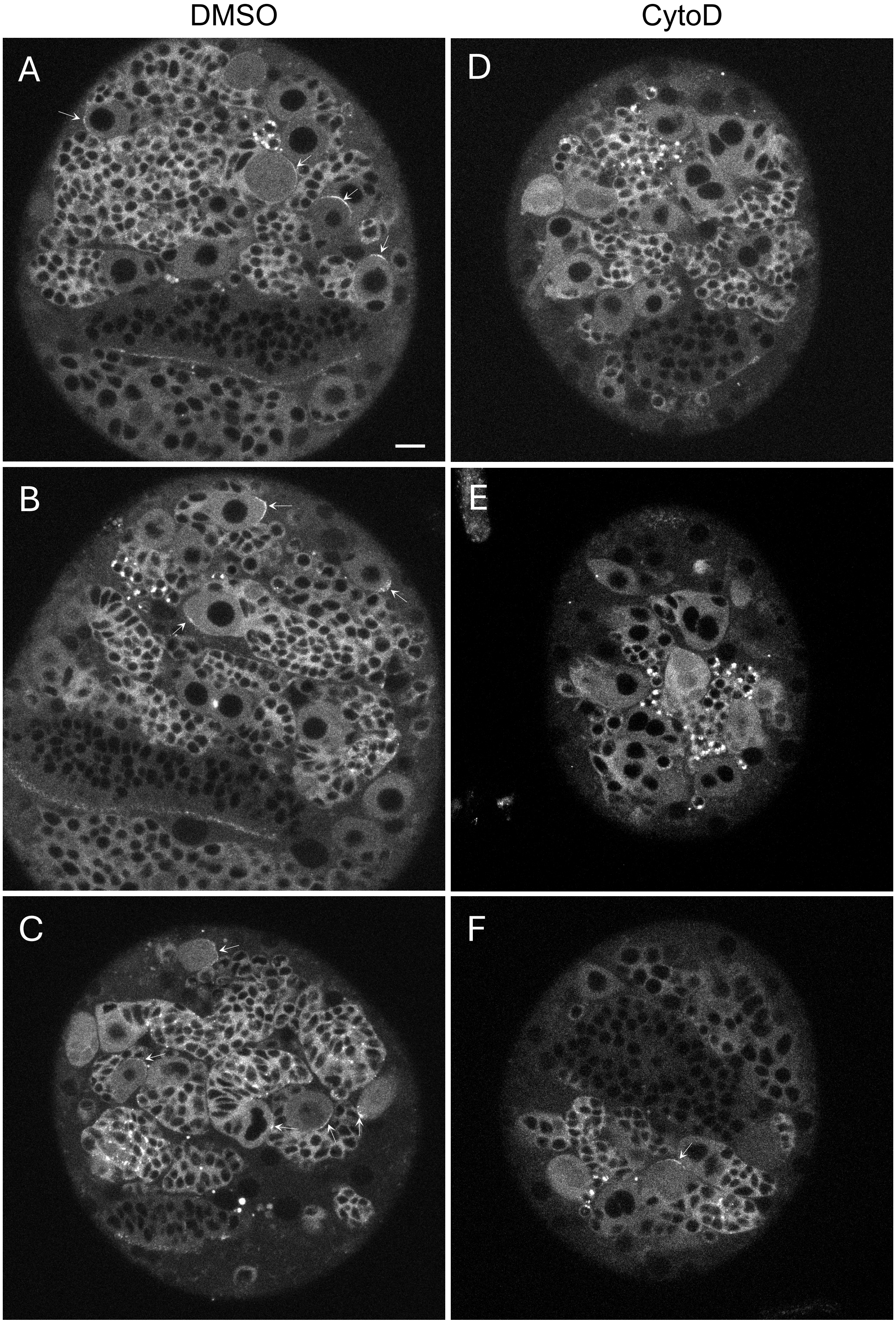

Fig. S2. Cytochalasin D (CytoD) treatment disrupts Kib polarization. Larval brains were treated for 1 hr with either DMSO (A-C) or 100 µM CytoD (D-F) before mounting and observing. Maximum projections of 3 optical sections are shown. In control brains (A-C), apically polarized Kib (arrows) can be observed in multiple NBs. In contrast, in CytoD treated brains polarized Kib is rarely seen. Note the prevalence of binucleate NBs in D-F, indicating that NBs continue to cycle in the presence of CytoD. Scale bar = 10 µm.

Biology Open • Supplementary information

A

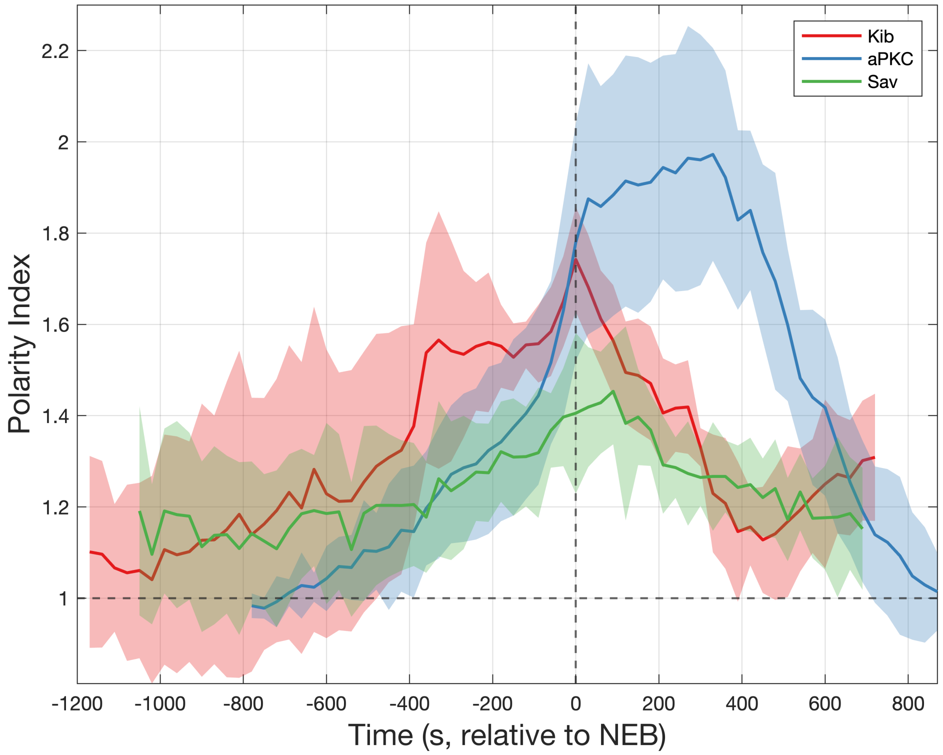

Fig. S3. Salvador polarization dynamics. A. The Polarity Index of multiple Ubi>Salvador-GFP neuroblasts plotted as a function of time. 7 neuroblasts are represented by the curve, and each timepoint represents the average of at least 3 neuroblasts. Shading represents one standard deviation from the mean. Kibra and aPKC curves reproduced from Fig. 2E for reference. Vertical dashed line marks time = NEB, and horizontal dashed line marks Polarity Index = 1.

Biology Open • Supplementary information

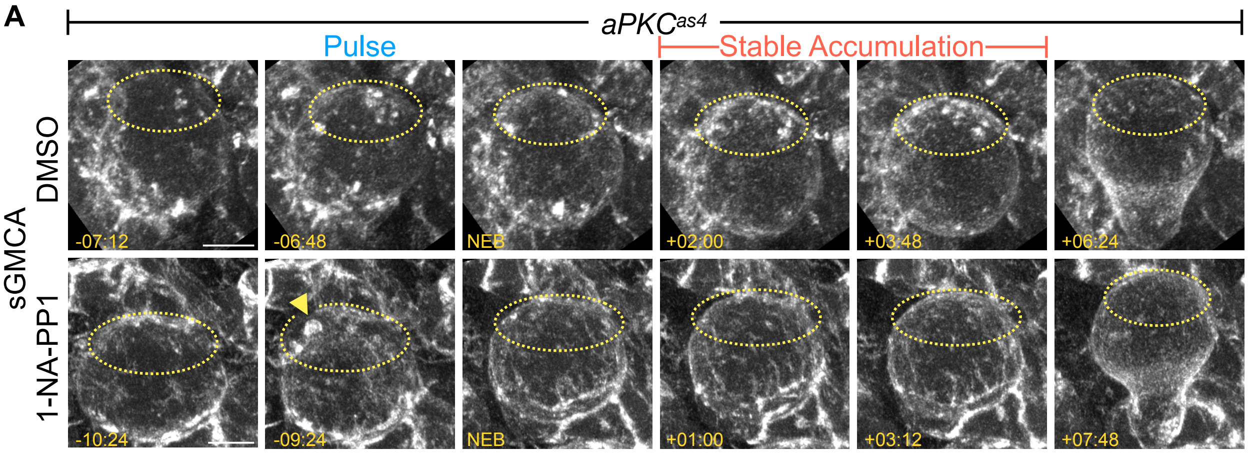

Fig. S4. aPKC regulates Actin dynamics in live neuroblasts. A. Mitotic neuroblasts expressing the F-actin reporter sGMCA (*spaghetti squash* driven, GFP, Moesin α-helical-coiled and actin binding site) from *aPKC*^{as4} animals treated with DMSO as a control or the aPKC^{as4} inhibitor 1-NA-PP1. Yellow dotted lines approximately demarcate the apical cortex. Yellow arrowhead indicates an example of a cortical bleb. Pulse refers to a single frame in which short-lived F-actin accumulation at the apical cortex was captured. Stable Accumulation refers to multiple frames in which long lived F-actin accumulation at the apical cortex was captured. Note the cortical Actin blebbing during the Pulse phase and diminished apical Actin accumulation during the Stable Accumulation phase in the treated neuroblast.

Table S1. List of chemical reagents

Name	Source	Identifier
1-NA-PP1	Cayman	Item # 10954
Cytochalasin D	Fisher (MP Biomedicals)	Cat # ICN15077101
Latrunculin A	Fisher (Enzo)	Cat # 501031800
JFX650	Gift from Janelia Research Campus	
JF646	Promega	GA1121

Table S2. List of *D. melanogaster* stocks

Name	Source	Identifier
insc>Gal4	Bloomington Drosophila Stock Center (BDSC)	8751
insc>Gal4; UAS-mCh-Tub	BDSC	25773
kib::YFP	Su et al., 2017	
Ubi>Kib-GFP	Tokamov et al., 2021	
Ubi>Kib-Halo	Tokamov et al., 2023	
Ubi>Kib$^{\Delta aPKC}$-GFP	Tokamov et al., 2023	
Ubi>Kib$^{858\text{-}1288}$-GFP	Tokamov et al., 2023	
Ubi>Sav-GFP	Aerne et al., 2015	
aPKC^{as4}	Hannaford et al., 2019	
Halo-Snap-aPKC	Erdmann et al., 2019	
RNAi of Kib	BDSC	28683
RNAi of Sav	BDSC	28006
UAS-LifeAct-Halo	BDSC	67625
GFP:wts	Rauskolb et al., 2014	
Dlg-GFP	FlyTrap (Buszczak et al., 2007)	CC01936
Baz-GFP	FlyTrap	CC01941
sGMCA	D. Kiehart (Edwards et al., 1997)	
Ubi>Mer-Halo	Tokamov et al., 2023	

Biology Open • Supplementary information

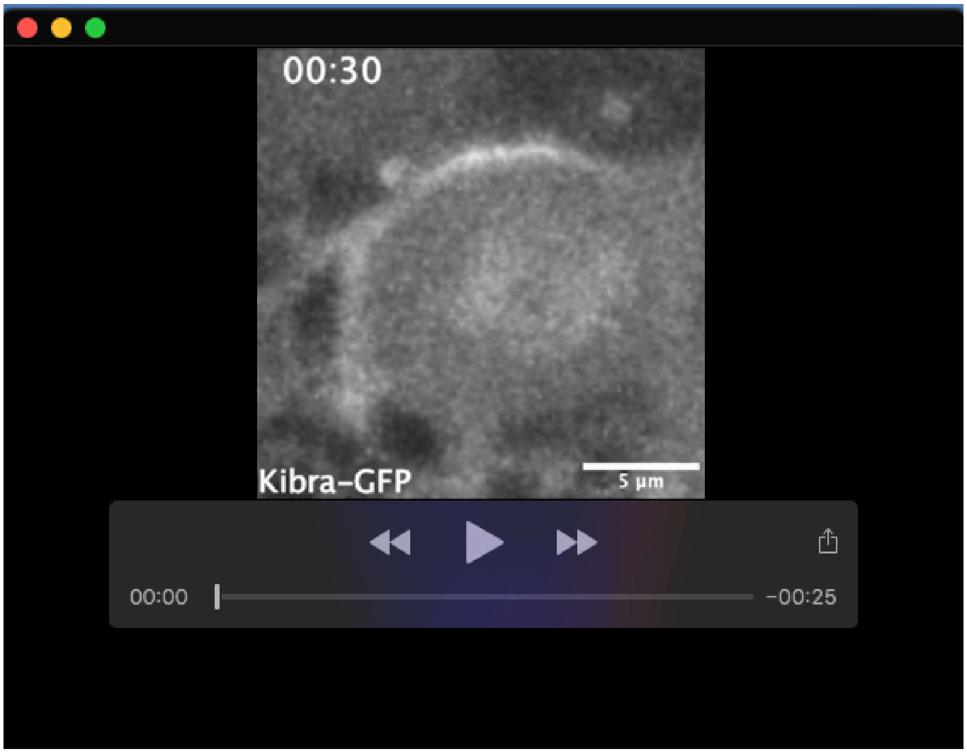

Movie 1 (related to Fig. 1B). Mitotic neuroblast expressing Ubi>Kib-GFP.

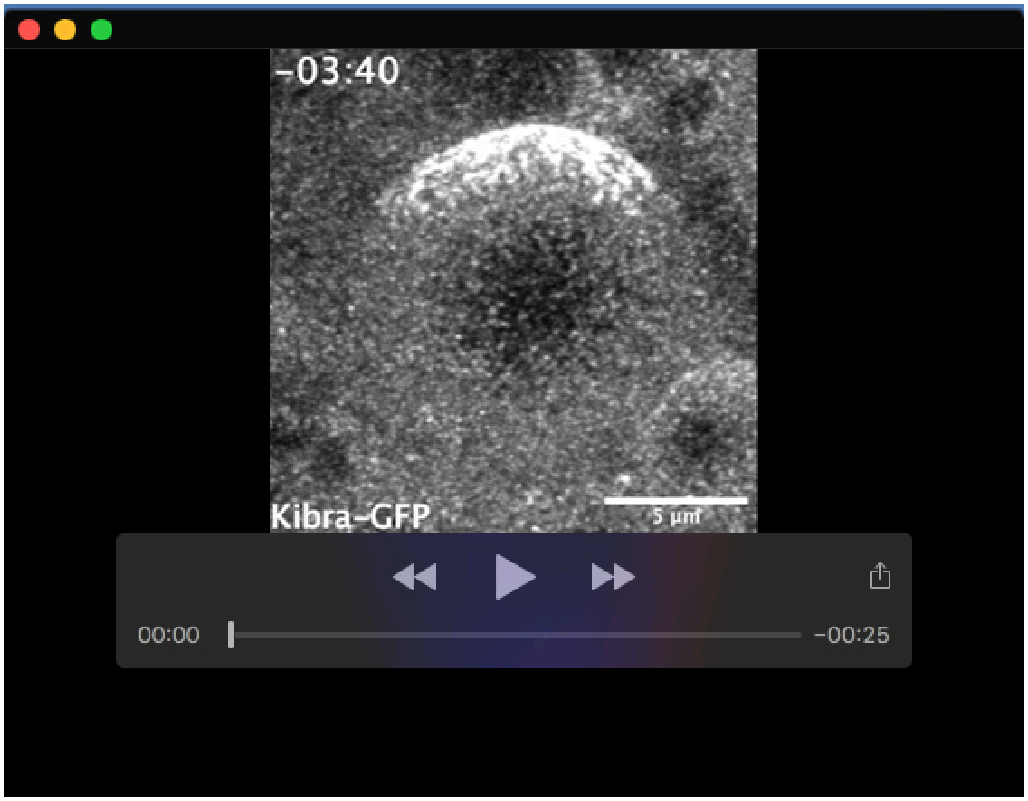

Movie 2 (related to Fig. 1C). Super-resolution view of the apical cortex of a mitotic neuroblast expressing Ubi>Kib-GFP in prophase.

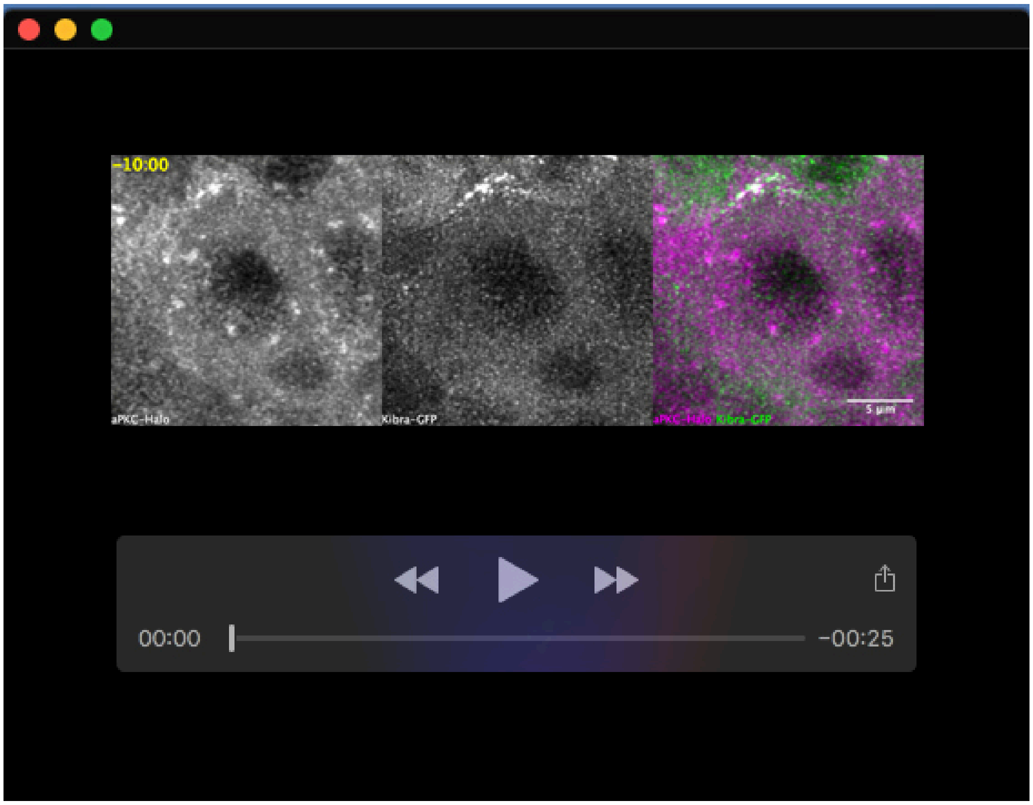

Movie 3 (related to Fig. 2A). Mitotic neuroblast coexpressing aPKC-Halo and Ubi>Kib-GFP.

Biology Open • Supplementary information

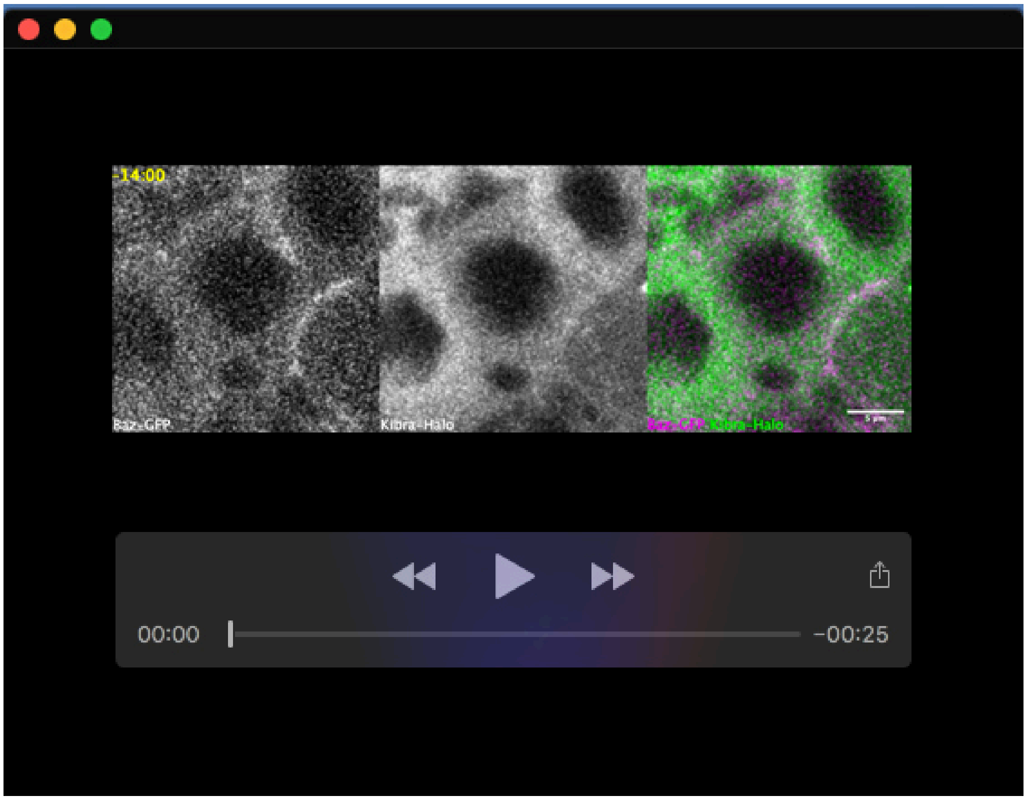

Movie 4 (related to Fig. 2B). Mitotic neuroblast coexpressing Baz-GFP And Ubi>Kib-Halo.

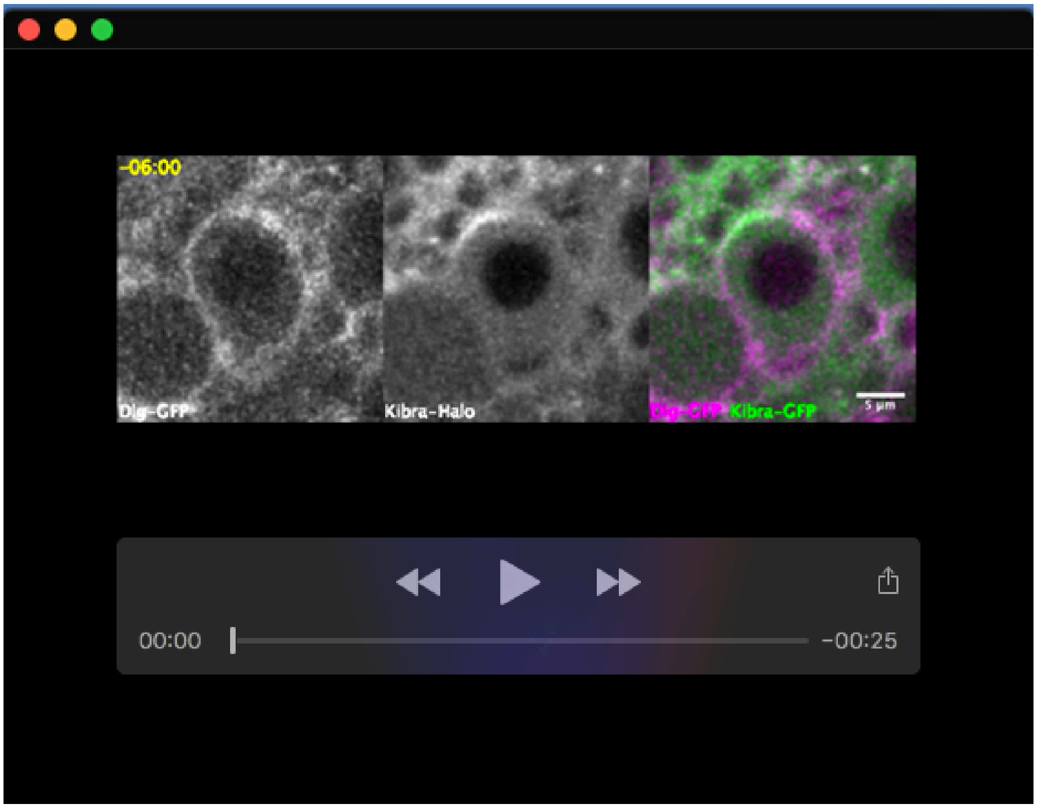

Movie 5 (related to Fig. 2C). Mitotic neuroblast coexpressing Dlg-GFP and Ubi>Kib-Halo.

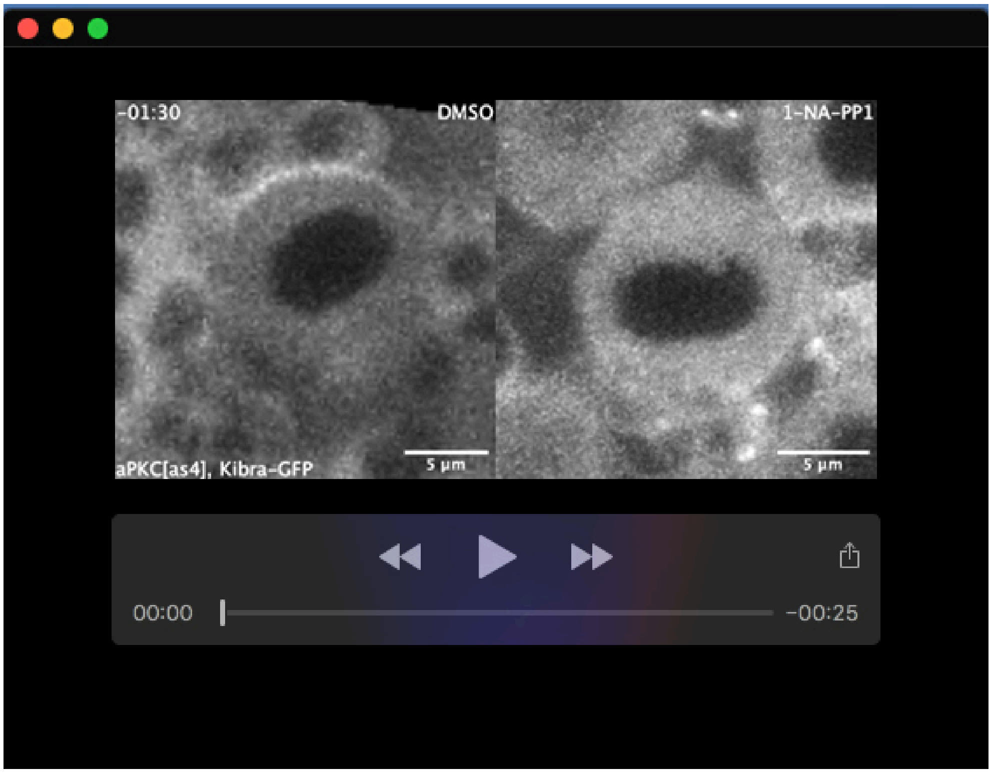

Movie 6 (related to Fig. 3A). Effect of acute aPKC inhibition with 1-NA-PP1 on Ubi>Kib-GFP polarization in *aPKCas4* neuroblasts.

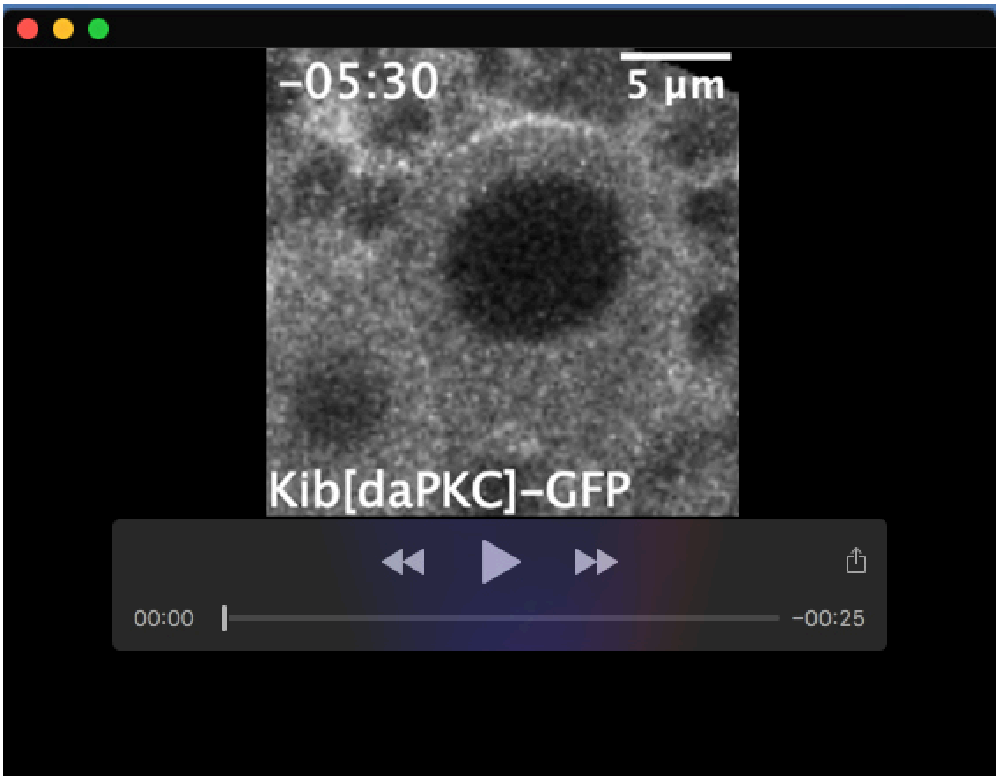

Movie 7 (related to Fig. 4B). Mitotic neuroblast expressing Ubi>Kib$^{\Delta aPKC}$-GFP.

Biology Open • Supplementary information

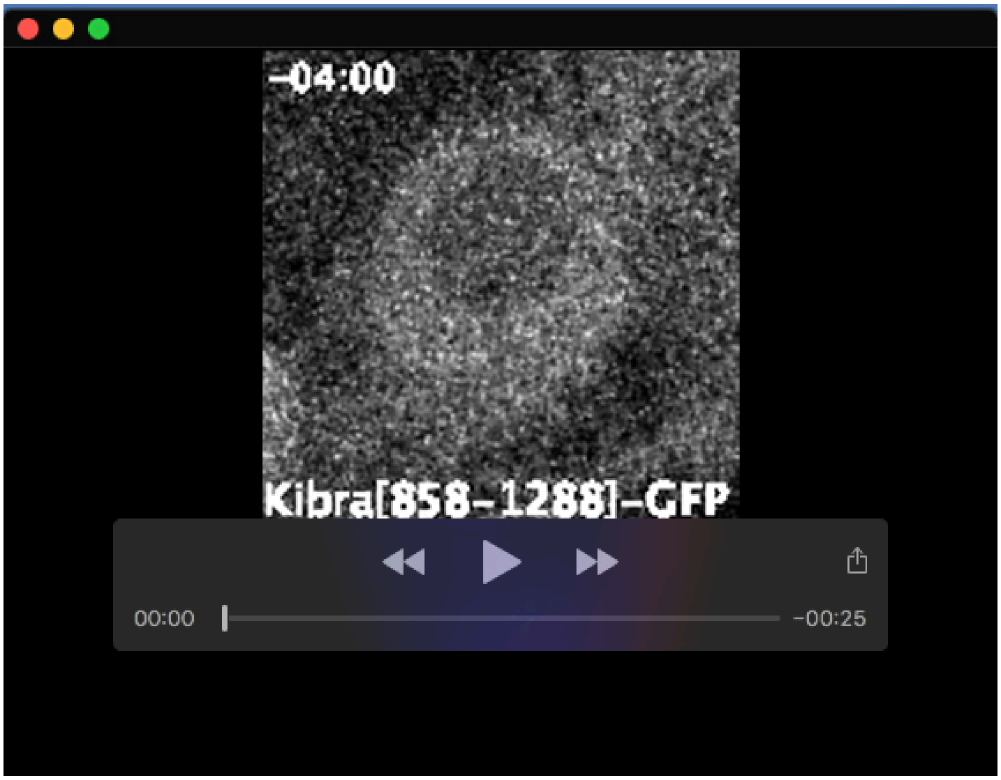

Movie 8 (related to Fig. 4C). Mitotic neuroblast expressing Ubi>Kib$^{858-1288}$-GFP.

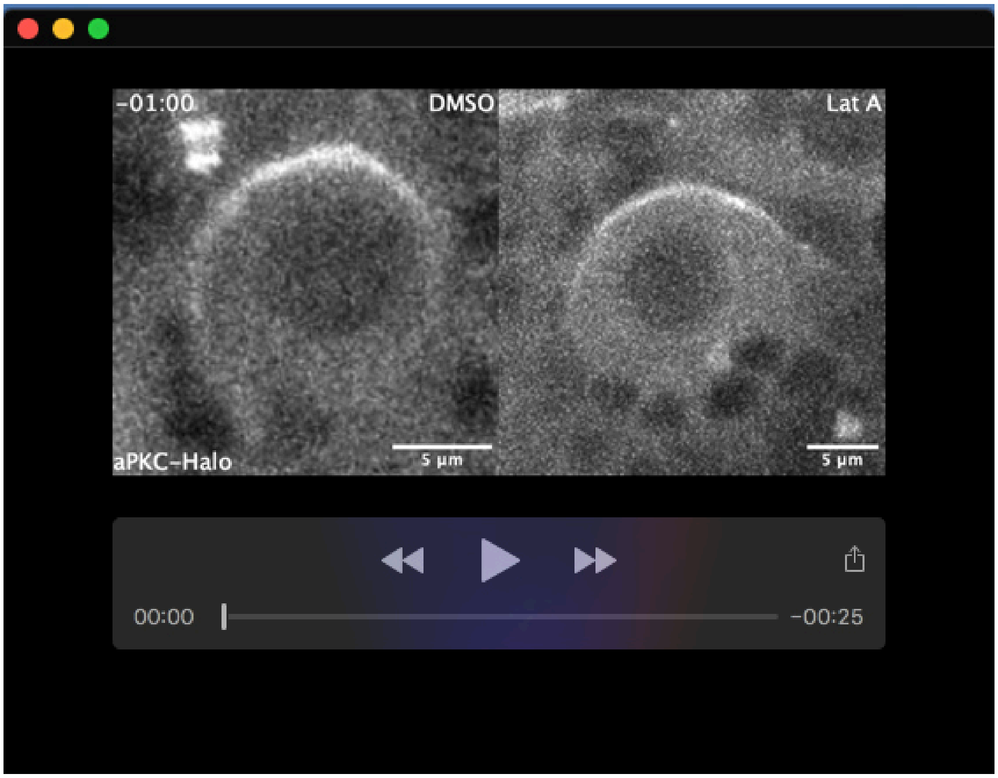

Movie 9 (related to Fig. 5A). Effect of F-actin inhibition with Latrunculin A on aPKC-Halo polarization. Same neuroblast as Movie 10.

Biology Open • Supplementary information

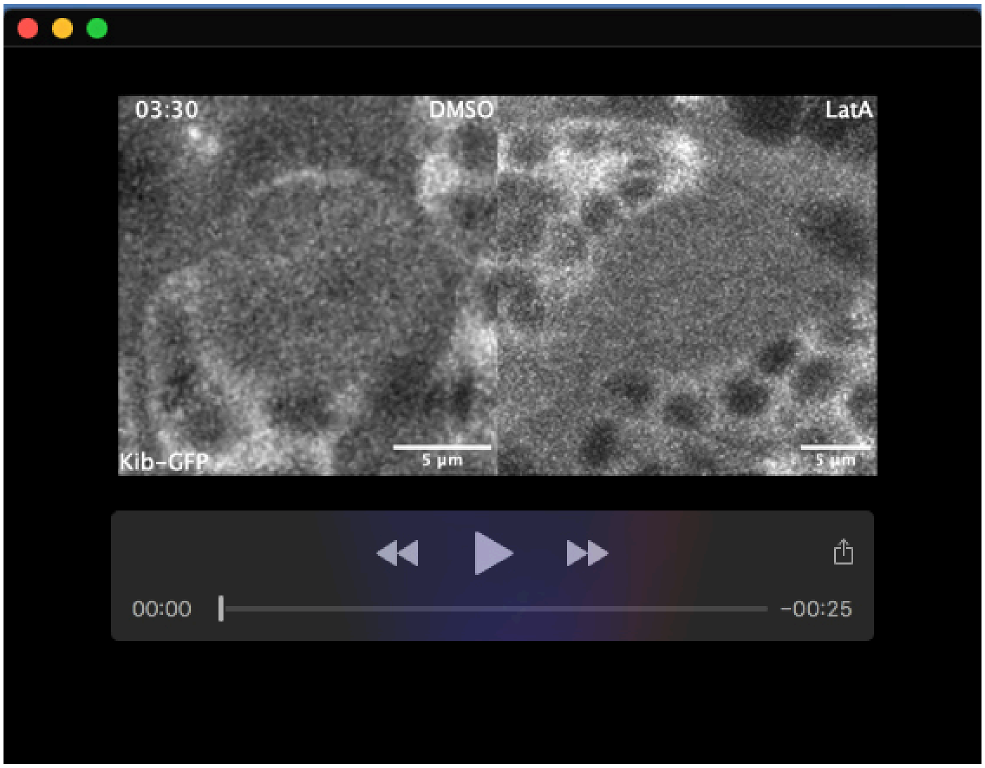

Movie 10 (related to Fig. 5B). Effect of F-actin inhibition with Latrunculin A on Ubi>Kib-GFP polarization. Same neuroblast as Movie 9.

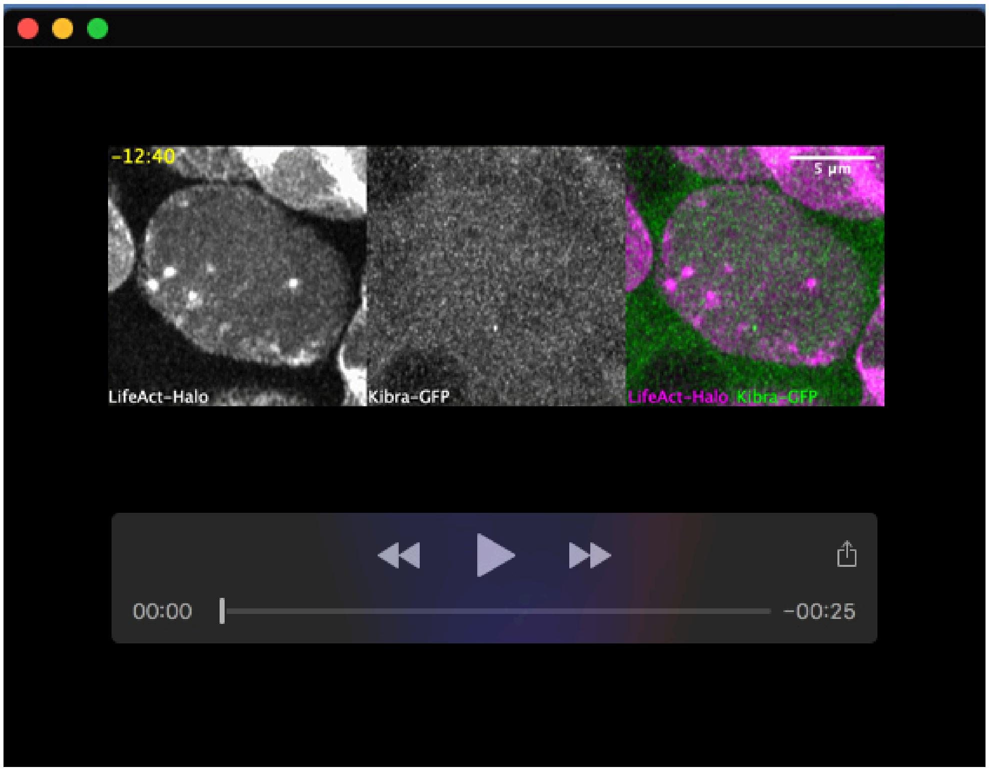

Movie 11 (related to Fig. 6A-C). Apical max projection of a mitotic neuroblast coexpressing LifeAct-Halo and Ubi>Kib-GFP. Top-down view.

Biology Open • Supplementary information

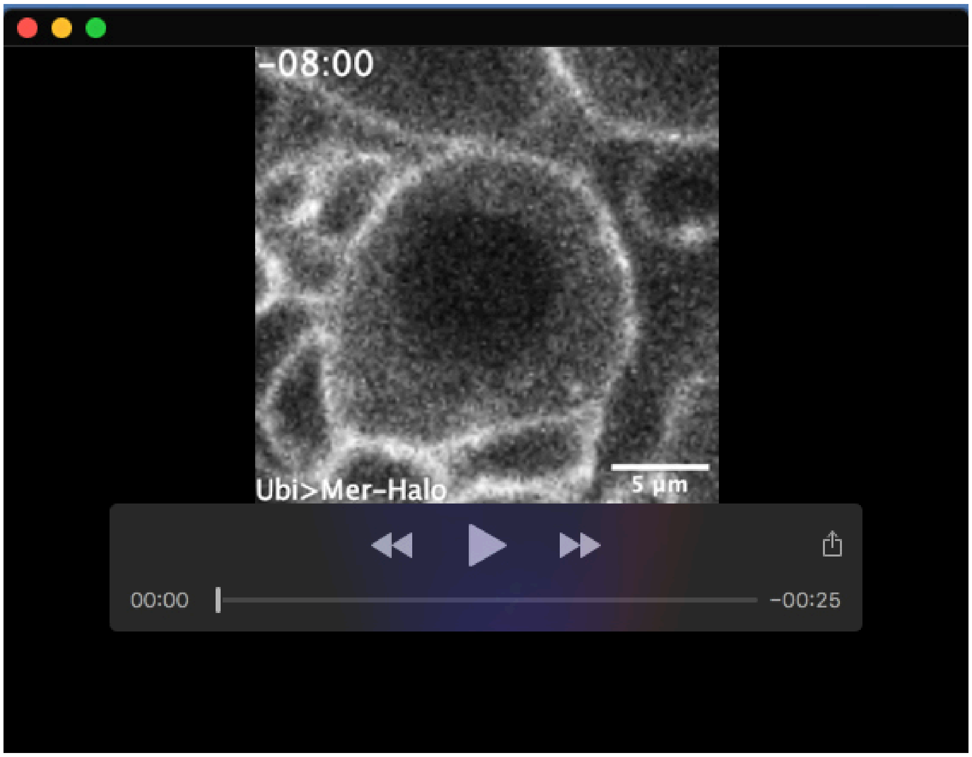

Movie 12. Mitotic neuroblast expressing Ubi>Mer-Halo. This view is of a single cortical slice.

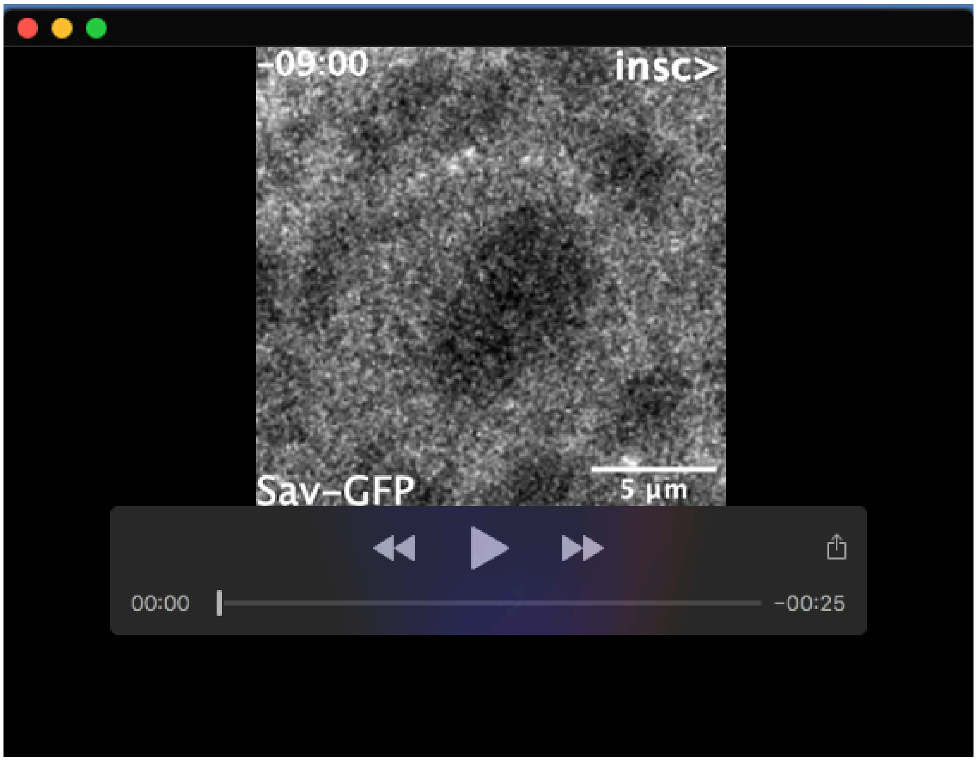

Movie 13 (related to Fig. 7A). Mitotic neuroblast expressing Ubi>Sav-GFP.

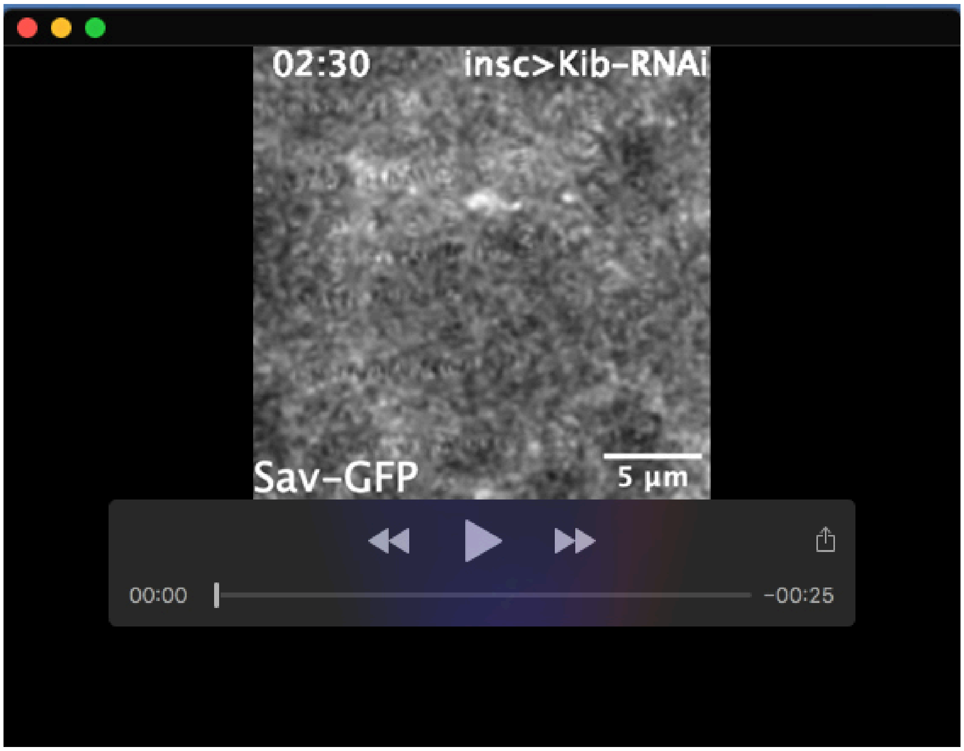

Movie 14 (related to Fig. 7A). Effect of Kib knockdown with insc>Kib-RNAi on Ubi>Sav-GFP polarization.

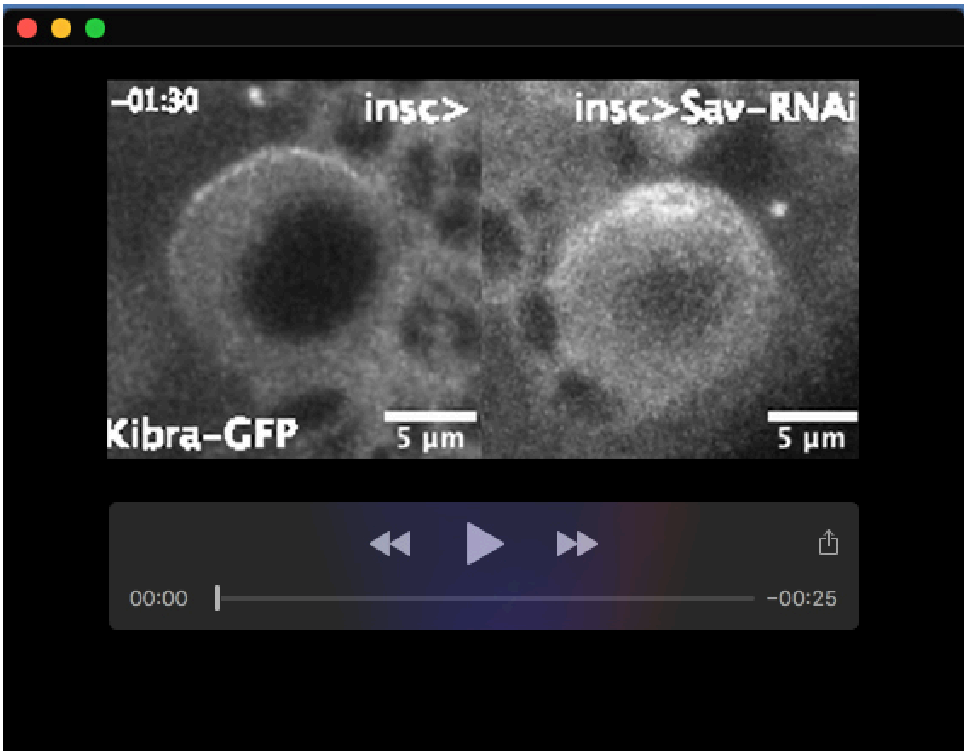

Movie 15 (related to Fig. 7B). Effect of Sav knockdown with insc>Sav-RNAi on Ubi>Kib-GFP polarization.

Biology Open • Supplementary information

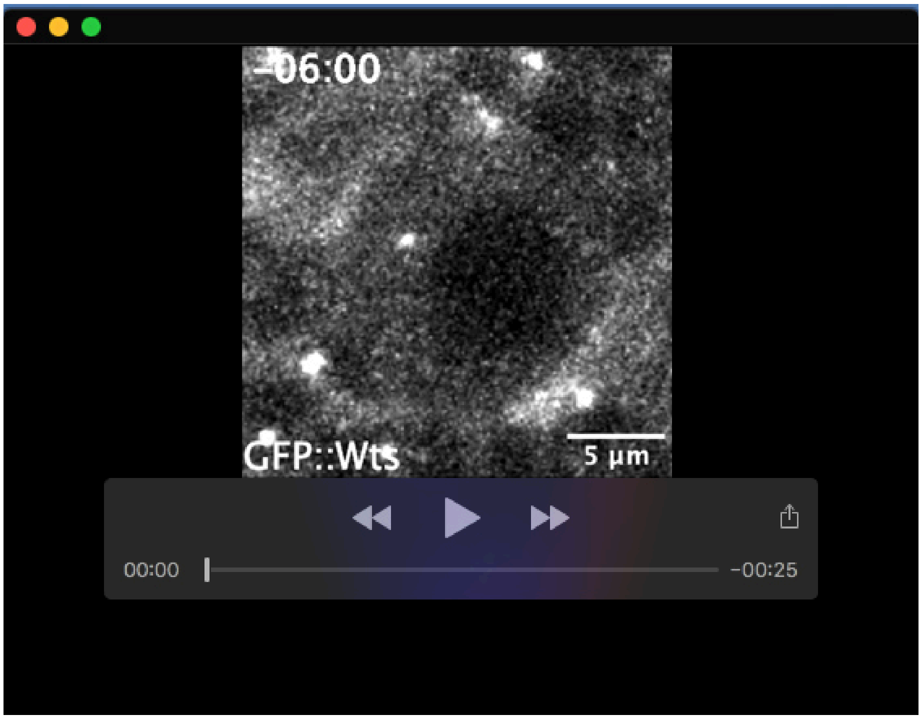

Movie 16 (related to Fig. 8A). Mitotic neuroblast expressing GFP:Wts.

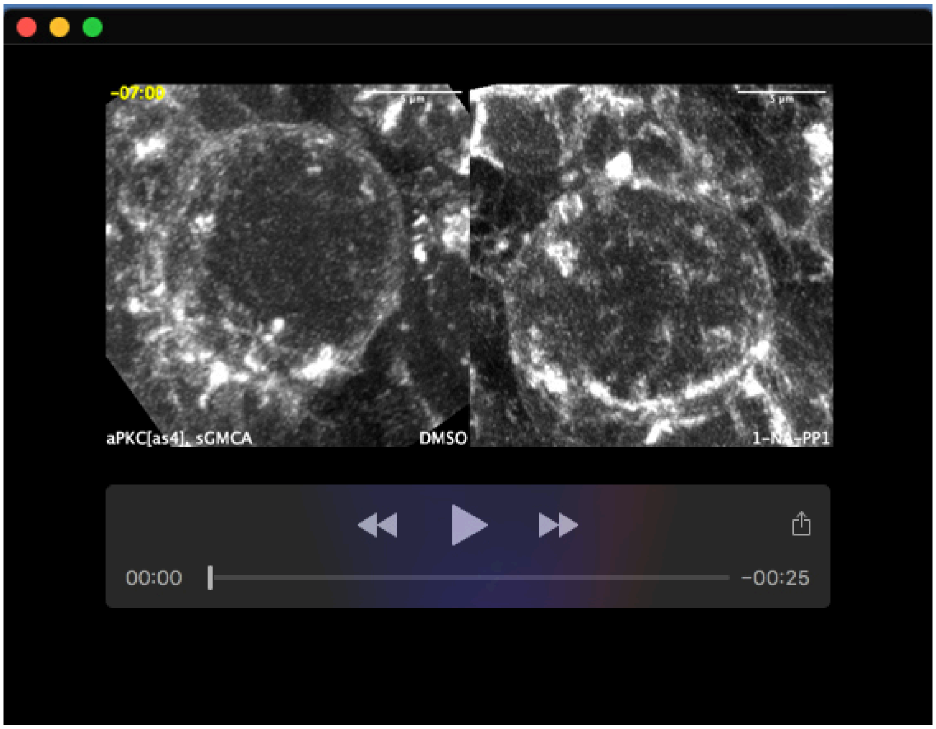

Movie 17 (related to Fig. S4A). Effect of acute aPKC inhibition with 1-NA-PP1 on cortical F-actin dynamics, visualized with the F-actin reporter sGMCA (*spaghetti squash* driven, moesin-alpha-helical-coiled and Actin binding site fused to GFP).
